# Neurokinin-1 Receptor (NK-1R) Antagonists as a New Strategy to Overcome Cancer Resistance

**DOI:** 10.3390/cancers14092255

**Published:** 2022-04-30

**Authors:** Marilina García-Aranda, Teresa Téllez, Lauraine McKenna, Maximino Redondo

**Affiliations:** 1 Research and Innovation Unit, Hospital Costa del Sol, Autovía A-7, km 187, 29603 Marbella, Spain; marilina@hcs.es (M.G.-A.); mclauraine@hcs.es (L.M.); 2 Instituto de Investigación Biomédica de Málaga (IBIMA), C/Dr. Miguel Díaz Recio, 28, 29010 Málaga, Spain; 3 Red de Investigación en Servicios de Salud en Enfermedades Crónicas (REDISSEC) and Red de Investigación en Cronicidad, Atención Primaria y Promoción de la Salud (RICAPPS), Instituto de Investigación Biomédica de Málaga (IBIMA), 29010 Málaga, Spain; teresatellez@uma.es; 4 Surgical Specialties, Biochemistry and Immunology Department, Faculty of Medicine, University of Málaga, 29010 Málaga, Spain

**Keywords:** cancer, tachykinin, tachykinin receptor, NK-1R, targeted treatment, drug repurposing, personalized medicine, resistance

## Abstract

**Simple Summary:**

Improving the response, quality of life and survival of oncologic patients through the design and application of the most appropriate treatment for each case is a great scientific challenge. Given the role of the axis formed by Substance P (SP) and its preferred receptor neurokinin-1 receptor (NK-1R) in cancer progression and resistance to oncologic treatments, in this review, we evaluate if the repurposing of aprepitant, which is a safe, efficient and marketed NK-1R antagonist, may be of help to overcome resistance to anticancer treatments.

**Abstract:**

Nowadays, the identification of new therapeutic targets that allow for the development of treatments, which as monotherapy, or in combination with other existing treatments can contribute to improve response rates, prognosis and survival of oncologic patients, is a priority to optimize healthcare within sustainable health systems. Recent studies have demonstrated the role of Substance P (SP) and its preferred receptor, Neurokinin 1 Receptor (NK-1R), in human cancer and the potential antitumor activity of NK-1R antagonists as an anticancer treatment. In this review, we outline the relevant studies published to date regarding the SP/NK-1R complex as a key player in human cancer and also evaluate if the repurposing of already marketed NK-1R antagonists may be useful in the development of new treatment strategies to overcome cancer resistance.

## 1. Introduction

### 1.1. The Challenge of Drug-Resistant Tumors

Cancer remains a major public health problem and a leading cause of death worldwide, accounting for more than 19 million new cases and nearly 10 million deaths in 2020 [1]. At the present time, one of the main challenges in the treatment of cancer patients is overcoming resistance, a clinical situation in which the tumor does not respond to treatment (intrinsic or primary resistance) or, although it initially responded, it ends up relapsing and progressing (secondary or acquired resistance).

Tumor progression is characterized by the sequential appearance of genetically altered cell subpopulations, which, under the selective pressure caused by anti-cancer treatments, promote the accumulation of irreversible changes and the proliferation of cell populations resistant to anticancer treatments [2]. This loss of therapeutic response of cancer cells against anti-cancer drugs, or multidrug resistance, can occur during or after treatment and may lead to the development of cross resistance to either structurally or mechanistically different chemotherapeutics.

**Figure 1:**
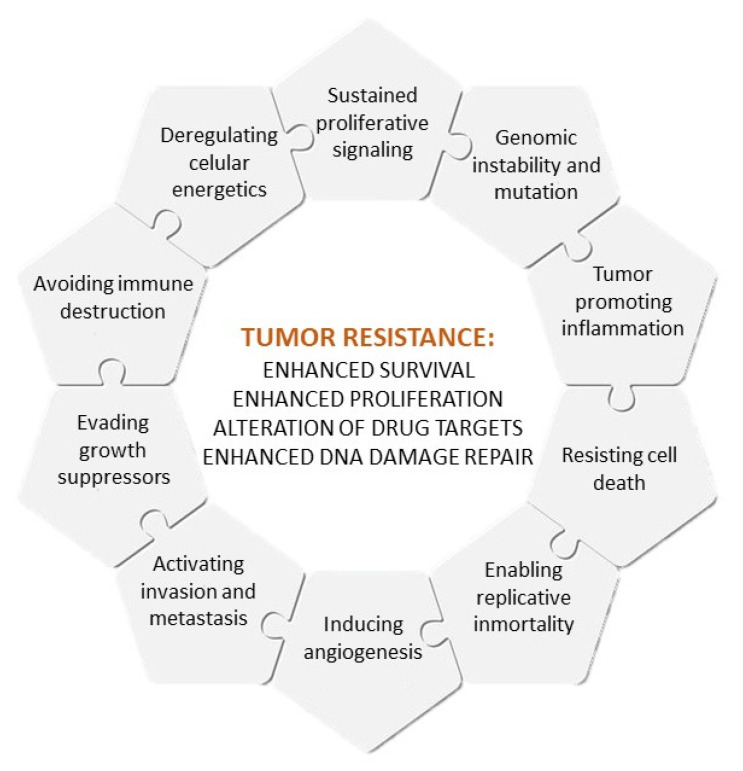
Hallmarks of cancer and causes of multidrug resistance.

The causes of multidrug resistance can include mechanisms as diverse as the elevated metabolism of xenobiotics, enhanced efflux of drugs, increased DNA repair capacity, genomic instability, reduced apoptosis or altered proliferation [3,4], which are, to some extent, related to the different hallmarks of cancer, as defined by Hanahan and Weinberg [5] (Figure 1). Nowadays, intrinsic and acquired multidrug resistance represent the main cause of treatment failure for patients with blood or solid tumors, being responsible for over 90% of deaths in oncologic patients treated with conventional chemotherapy or novel targeted treatments [3].

Although recent advances in cancer research have allowed for the development of targeted treatments for resistant tumors, most of them have failed in clinical stages, mainly due to their low specificity and high toxicity [6]. For this reason, cancer research is nowadays focused on the development of more effective and safe targeted therapies.

As for other drugs, the development of new therapeutic agents for cancer treatment requires the validation of drug safety and efficacy, which involves multiple phases, from the earliest basic investigation to clinical testing and final authorization. Since this long-term process of authorization of new drugs for cancer treatment, from bench to bedside, is associated with a high risk of failure, timeframe and overall costs [7], the use of known marketed drugs, which have already been assessed for safety and efficacy, for new therapeutic purposes, or drug repurposing, has gained increasing popularity in recent times as a strategy to enhance new pharmaceutical development with rapid clinical translation [8], having proved to shorten the development process by 3–5 years and to increase the success rates from 10% to 25% [7]. So much so that up to 30% of all the current drugs and vaccines approved in recent years by the US Food and Drug Administration (FDA) are repurposed drugs [9], some of which are already being tested to see if they can be useful for the treatment of resistant tumors.

G-protein-coupled receptors (GPCRs) are the largest family proteins targeted by approved drugs, accounting for approximately 17% of FDA agents against human proteins [9,10]. In 2003, aprepitant (EMEND, Merck Sharp & Dohme B.V.) became the first tachykinin-1 receptor (TAC1R, aka neurokinin-1 receptor NK-1R) antagonist approved for the prevention of acute and delayed chemotherapy-induced nausea and vomiting, a side-effect that can affect more than 99% of patients treated with cisplatin [11].

Studies on cancer research also reported that both NK-1R and its preferred ligand (neurokinin 1, NK-1 aka substance P, SP) are overexpressed in a wide variety of malignancies, including leukemia, glioblastoma, astrocytoma, neuroblastoma, melanoma, breast, ovarian, prostate, lung, pancreas and thyroid cancer [12,13], with a role in different driving agents to resistance, such as angiogenesis, cancer cell proliferation, migration and metastasis [12,13,14,15,16]. As a result of a recent clinical report [17] and different in vitro and in vivo studies showing that NK-1R antagonists can exert an antitumor, antiproliferative, anti-survival, antiangiogenic and antimetastatic effect [14,16,18], the inhibition of the NK1-R/SP axis has been proposed as a promising therapeutic approach to battle cancer and cancer resistance [19,20,21], justifying additional investigations that support the reprofiling of marketed NK-1R antagonists, such as aprepitant, as therapeutic agents for cancer treatment, in addition to their use in clinical practice as antiemetic.

### 1.2. Tachykinins

Since the identification of substance P in 1931, a number of short, highly conserved, bioactive peptides, called tachykinins, have been isolated and investigated, constituting at present one of the largest families of neuropeptides. In humans, tachykinins are expressed throughout the nervous and immune system, with an important role in the regulation of a wide range of physiological processes that include inflammation, nociception, smooth muscle contractility, epithelial secretion and cell proliferation in the nervous, immune, gastrointestinal, respiratory, urogenital and dermal systems [22].

All members of this family are structurally related peptides characterized by an amidated C-terminal, whose deamidation suppresses peptide activity [23], and a conserved C-terminal sequence -Phe-Xaa-Gly-Leu-Met-NH2, where Xaa represents a hydrophobic amino acid residue [24,25] (Table 1) required for the activation of the corresponding receptor (tachykinin receptor 1/NK-1R, 2/NK-2R or 3/NK-3R) [22], which is responsible for signal transmission from the extracellular environment to the cytoplasm.

In humans, tachykinins are encoded by three different genes, *TAC1* (tachykinin precursor 1), *TAC3* (tachykinin precursor 3) and *TAC4* (tachykinin precursor 4) (Table 1):

- The transcription of the *TAC1* gene (NCBI Gene ID: 6863) produces the pre-protachykinin-A (*PPTA*)-*mRNA*, which is converted into one of four splice variants coding for a pro-tachykinin polypeptide that contains NK-1 [29], Neurokinin A (NKA, formerly known as substance K) and the NH2-terminally extended forms of NAK neuropeptide K (NPK) and neuropeptide gamma (NPγ) [22,26,30]. These peptides function as neurotransmitters by interacting with nerve receptors and smooth muscle cells [30].
- *TAC3* (NCBI Gene ID: 6866) encodes a preprotein that is further cleaved to generate a mature secreted neuropeptide (neurokinin B, NKB). NKB is primarily expressed in the central and peripheral nervous systems and functions as a neurotransmitter [31]. NKB is a critical central regulator of gonadal function and its alterations are mainly associated with hypogonadotropic hypogonadism [32].
- Finally, *TAC4* (NCBI Gene ID: 255061) produces endokinins (EK) A, A/B, C and D as well as hemokinins [12,28], which are associated with the hematopoietic system and lymphocyte B maturation [12]. *TAC4* gene products are thought to regulate different peripheral endocrine and paracrine functions, including blood pressure, the immune system and endocrine gland secretion [33].

### 1.3. Tachykinins and Tachykinin Receptors in Human Disease and as Pharmacological Targets

Tachykinins have been associated with different pathological processes, including neurogenic diseases, such as schizophrenia, Alzheimer’s disease and Huntington’s disease [34], as well as acute and chronic inflammation and pain, fibrosis, affective and addictive disorders, functional disorders of the intestine and urinary bladder, infection or cancer [22]. Since tachykinin signaling on targeted cells is mediated by tachykinin receptors, most therapeutic approaches are designed to block ligand–receptor interactions.

#### 1.3.1. Tachykinins and Tachykinin Receptors in Human Disease

- Respiratory disorders

Both SP and NKA can be released from airway nerves after noxious stimulation [35], and have a role in respiratory functions, such as the regulation of airway smooth muscle tone, vascular tone and mucus secretion, and immune functions [35]. The overexpression of NK-1R and NK-2R receptors is usually found in inflammatory airway diseases, such as bronchial asthma or chronic obstructive pulmonary disease [35,36], with a role in bronchoconstriction, airway hyperresponsiveness and airway inflammation caused by allergic and nonallergic stimuli, which has prompted the development of both selective and dual-selective NK-1R/NK-2R antagonists that have entered clinic studies with promising results [36,37].

- Smooth muscle disfunction:

NK-2R is predominantly expressed on smooth muscle, with a role in the contraction of the intestinal, genito-urinary and respiratory tracts. The NK-2R antagonist MEN11420 (nepadutant) has demonstrated, both in preclinical and clinical studies, to be a well-tolerated and a potent, selective and competitive NK-2R inhibitor with an effective and long-lasting anti-spasmogenic effect without affecting basal gastrointestinal motility [38].

- Central nervous system disorders:

Non-peptide antagonists of NK-3R, which is mainly expressed in the central nervous system, have already shown in preclinical studies their potential utility as a strategy for the treatment of central nervous system disorders [39], such as schizophrenia, major depressive disorder, panic attacks and Parkinson’s disease [39].

Given the role of the SP/NK-1R axis in neuroinflammation associated with different bacterial, viral, parasitic and neurodegenerative diseases of the central nervous system, NK-1R antagonists have recently been proposed as a promising therapeutic agent for the treatment of neuroinflammation [40].

- Hormonal disorders:

Inactivating mutations in both NKB or its preferred receptor NK-3R have been associated with low gonadotropin-releasing hormone (GnRH) pulse frequency [41]. Both preclinical and clinical studies (phase I clinical trial EUDRACT 2013-004314-17) have shown that the oral administration of the NK-3R antagonist ESN364 is well-tolerated and selectively modulates gonadotropin secretion, which may be of help in the treatment of women’s health disorders with a low risk of menopausal-like adverse events [41].

#### 1.3.2. Marketed Tachykinin Receptor Antagonists

SP roles in both health and disease have motivated intense research by the pharmaceutical industry to develop selective tachykinin receptor agonists that could be of help in the treatment of human disorders.

Concretely, SP is associated with physiological processes as diverse as hematopoiesis, wound healing, microvasculature permeability, neurogenic inflammation, leukocyte trafficking, cell survival and metastatic dissemination [12], as well as with the regulation of biological processes, such as the dilatation of the arterial vascular system, neuronal survival and degeneration, respiratory function, sensory perception, movement control of gastric motility, salivation, micturition, pain and depression [13].

On March 2006, the FDA approved EMEND^®^ (aprepitant) as the first NK-1R antagonist for the prevention of acute and delayed nausea and vomiting associated with initial and repeat courses of highly and moderately (approved on 28 October 2008) emetogenic cancer chemotherapy, as well as for the prevention of post-operative nausea and vomiting (approval on 30 June 2006) in adult patients [42].

Contrary to other previously developed drugs, aprepitant also included a novel nanoparticle formulation to optimize oral absorption that allows for its administration as a water-soluble phosphoryl prodrug form (Ivemend, fosaprepitant) suitable for intravenous administration [43]. Aprepitant selectively blocks the activation of NK-1R in vomiting centers within the central nervous system; thus, since approximately 25% and 50% of patients experience acute or delayed chemotherapy-induced nausea and vomiting, respectively [11], the marketing authorization of this non-peptide NK-1R antagonist represented a great step forward in enhancing the quality of life of oncologic patients who must undergo multiple cycles of chemotherapy [43].

In 2014, the US FDA approved the combination of NK-1R antagonist netupitant plus the 5-HT_3_R receptor antagonist palonosetron (Akynzeo; Eisai) for the prevention of acute and delayed nausea and vomiting associated with initial and repeated courses of cancer chemotherapy [44].

More recently, in 2015, the FDA also approved aprepitant for patients of 6 months of age and older [42] as well as Varubi^©^ (rolapitant) tablets [45] and rolapitant injectable emulsion [46], as other non-peptide SP/NK-1R antagonists with the same indication as aprepitant/fosaprepitant, but with a better safety profile and lower incidence of adverse events [47].

#### 1.3.3. Aprepitant as Candidate for Drug Repurposing

Aprepitant is a safe clinical drug that, in addition to its antiemetic effect, has also demonstrated to have anxiolytic, antidepressant, anti-inflammatory and analgesic effects [16] that can increase pain tolerance in surgical procedures [48]. Furthermore, during the last decade, different in vitro and preclinical studies have suggested that aprepitant could also be prescribed for the treatment of nausea, analgesia, migraine, asthma, urinary incontinence or gastrointestinal disorders. Although most clinical studies have failed to demonstrate activity for such conditions, there is evidence of its value for the treatment of alcohol dependence [49], nausea or severe pruritus associated with the use of biological agents in oncologic patients [50].

In the field of cancer research, multiple preclinical studies have demonstrated the main role of SP/NK-1R axis in inflammation and tumor environment [16], as well as in the different hallmarks of cancer, such as resisting cell death, inducing angiogenesis and promoting cell proliferation and migration [51]. Different groups have also proved the antitumor effect of NK-1R antagonists both in vitro and in vivo [49], and have also suggested the potential utility of these compounds to reverse chemoresistance [21].

Furthermore, although some studies suggest that aprepitant could interfere with the action of chemotherapeutic drugs by inhibiting the CYP3A4 enzyme involved in the clearance of such substances, it has also been show that concomitant administration of aprepitant does not change pharmacokinetics or the toxicity of standard doses of docetaxel in cancer patients [52], which, together, supports the need for further investigation to explore the potential utility of aprepitant as a novel therapeutic approach to improve current anticancer strategies.

## 2. Substance P/Neurokinin-1 System as a Target for Cancer Treatment

### 2.1. NK-1 Receptor

The implication of NK-1R overexpression in the promotion of angiogenesis, proliferation and metastasis in hematological as well as in solid malignancies, such as melanoma, breast, gastric, liver, colon and pancreatic cancer [53], and the activity of NK-1R antagonists as antiproliferative, anti-survival, antiangiogenic and antimetastatic in both in vitro and in vivo models have turned this receptor into a promising target for cancer treatment.

Human NK-1R represents the main receptor for the tachykinin peptide family [12] with a range order of affinity to SP, followed by NKA and NKB [54] (relative affinities for the NK-1R are 100 and 500-fold lower than that of SP, respectively) [55,56].

This receptor is encoded by the *TACR1* gene, whose 5’ flanking region has several conserved gene promoter regulatory elements, such as a cAMP responsive element (CRE), Activating Protein-1 (AP-1), AP-2 and AP-4, Nuclear Factor kB (NF-kB) and Octamer Binding Protein 2 (OCT-2) binding sites [56]. *TACR1* gene produces two spliced isoforms: the predominant form is a full-length 407 amino acid membrane protein, which is mainly expressed in the central and peripheral nervous systems [57], and a truncated form, which is expressed in different tissues and cells, such as monocytes, NK cells and T cells [57]. The truncated form lacks 96 residues at the C-terminus, which partially disrupts signaling motifs without affecting the SP binding domain [57].

As in the cases of NK-2R and NK-3R, NK-1R is a G-protein-coupled receptor consisting of one extracellular amino-terminus, seven hydrophobic α-helical-transmembrane domains, three intracellular loops, three extracellular loops and a cytoplasmic carboxyl tail [54]. The second and third membrane domains are involved in agonist and antagonist binding and the third cytoplasmic loop is required for G-protein interactions [12,56]. Specifically, studies show that, although NK-1R can couple to both G_q_ and G_s_ protein subtypes, which are responsible for signal transduction, this receptor presents a coupling preference to G_q_ over G_s_ [58].

**Figure 2:**
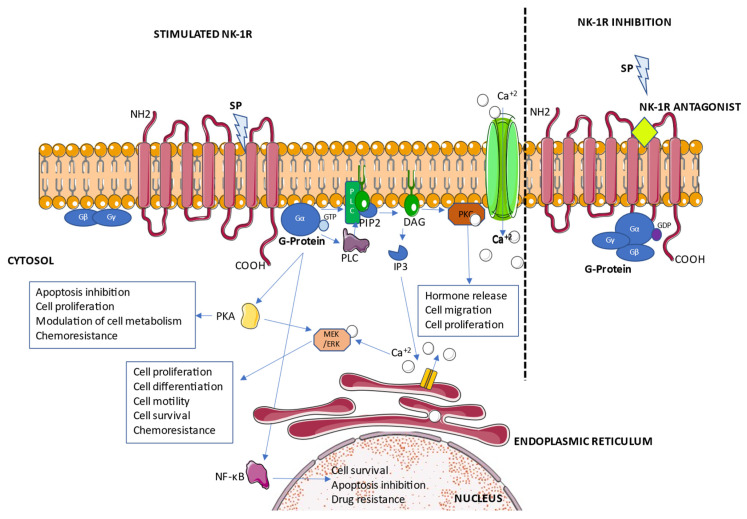
NK-1R signaling pathways related with chemoresistance.

Upon NK-1R stimulation by the agonist, the receptor adopts an active-state conformation that promotes the replacement of the GDP bound to the G protein alpha subunit by GTP, followed by the dissociation of the G protein subunits into the GTP-bound alpha subunit and a beta-gamma dimer, both of which remain anchored to the plasma membrane, but without interacting with the NK-1R [59] (Figure 2).

NK-1R antagonists block the access to the receptor binding site, hindering G-protein activation. In the case of aprepitant and netupitant, they behave as pseudo-irreversible antagonists, causing the long-lasting inhibition of receptor activation due to slower dissociation kinetics as well as by causing conformational changes of NK-1R that directly interfere with SP binding [58].

### 2.2. NK-1 Receptor Signaling Pathways with a Role in Cancer

As a result of G-protein stimulation by NK-1R, phospholipase C (PLC) enzymes are activated, promoting the hydrolysis of membrane phospholipid PtdIns(4,5)P2 (PIP2) to produce two second messengers: diacylglycerol (DAG), which activates protein kinase C (PKC) and the influx of extracellular calcium ions (Ca^+2^) through calcium channels down the electrochemical gradient [54], and inositol triphosphate (IP3), which promotes the release of Ca^+2^, a major second messenger crucial to rule the fate of cells, from endoplasmic reticulum to the cytoplasm [29] (Figure 2).

As a result of the increased Ca^+2^ concentration in the cytoplasm, different pro-survival and proliferative signaling pathways, such as those controlled by Mitogen-Activated Protein Kinase/Extracellular Signal-Regulated Kinases (MEK/ERK), are activated. The involvement of NK-1R in the positive regulation of cytosolic Ca^+2^ concentrations [56,60] is critical to a wide variety of vital processes, such as cell communication, migration, immune activation, cell death or proliferation [60,61], all of which can be deregulated and exploited by cancer cells [61,62], especially by those presenting NK-1R overexpression, the reason why this receptor represents a promising target for the treatment of multidrug resistant tumors.

On the other hand, NK-1R is co-expressed, co-localized and can crosstalk and transactivate epidermal growth factor receptor (EGFR) phosphorylation [63]. As a result of preclinical and clinical evidence, EGFR has become a validated target for cancer therapy in recent years [63] and, thus, NK-1R inhibitors represent potential therapeutic target for lung cancer treatment as well as to overcome resistance to EGFR inhibitors.

NK-1R interaction with its SP also derives to the phosphorylation of p21-activated kinases (PAK), serine/threonine kinases key regulators of cancer cells’ signaling networks [64], which activate different signal cascades, including Mitogen Activated Protein Kinases (MAPK) and Phosphatidylinositol 3-kinase/Protein kinase B (PI3K/Akt) pathways. Both of these pathways are considered to be main causes of chemoresistance in cancer therapy by inhibiting apoptosis, stimulating proliferation and modulating cell metabolism [65], and, hence, one of the major targets for the treatment of multidrug resistant tumors. PAKs also stimulate the activation of MEK/ERK, which are pivotal players of chemoresistance in cancer, by regulating the formation of MEK1/ERK complexes [66]. Indeed, in addition to promoting cell proliferation, ERK1/2 has an important role in the adaptation of cancer cells to chemotherapy, having been reported to promote resistance in endometrial, gastric, colon, breast, ovarian, liver, esophageal, prostate, non-small and small cell lung cancers, osteosarcoma, neuroblastoma, glioma and T-cell acute lymphoblastic leukemias [67]. Specifically, the SP interaction with NK-1R stimulates the formation of a multiprotein scaffolding complex close to the plasma membrane in vitro, containing the internalized NK-1R, β-arrestin, the proto-oncogene tyrosine-protein kinase Src and the extracellular signal-regulated kinases 1 and 2 (ERK1/2), which are subsequently internalized into endosomes. This scaffolding complex would facilitate the nuclear translocation of activated ERK1/2 and foster the proliferative and antiapoptotic effects of NK-1R activation [68].

Given the important role of ERK1/2 in promoting an immune-evasive phenotype in colon, breast, prostate, liver, non-small cell lung cancer, pleural malignant mesothelioma, gastrointestinal sarcoma, Lewis lung carcinomas, melanoma and glioblastoma cancer models [67], targeting SP/NK-1R axis could also be a promising target to enhance the response to anti-cancer immunotherapies.

Moreover, SP interaction with NK-1R promotes the activation of Nuclear Factor kappa-light-chain enhancer of activated B cells (NF-kB), a transcription factor that has a role in activating cell survival, DNA transcription and the production of tumor-associated cytokines and mediators, such as IL-1β, IL-6, Tumor Necrosis Factor-alpha (TNF-α), Macrophage Inflammatory Protein-1beta (MIP-1beta) and interferon gamma (IFN-γ) [29,57,69]. As for PAK, NF-κB has been proven to inhibit apoptosis, promote cancer development and progression as well as to induce drug resistance in cancer cells [70,71], having been identified as potential and promising targets for drug discovery in cancer. The potential utility of NK-1R antagonists to improve the efficacy of anti-cancer immunotherapies would be more pronounced in NK-1R overexpressing tumors, since SP can also act as a chemoattractant for human monocytes, being able to amplify the inflammatory response by promoting immunoglobulin production and the secretion of proinflammatory mediators and cytokines from lymphocytes, monocytes, macrophages and mast cells, and thus, stimulating both lymphocyte proliferation and recruitment [56].

Finally, it should be emphasized that, since the truncated NK-1R form has a 10-fold less binding affinity to SP than the full-length, which is related to a diminished calcium response [29], the ability of SP to induce the expression of antiapoptotic genes and protect against apoptosis via ERK1/2 activation would depend on the expression of the full length or the truncated variant of the NK-1R receptor [68] and, therefore, the study of the type of NK-1R variant expressed in tumor cells could be of interest as a criterion for patient selection in eventual clinical studies in which the utility of SP antagonists as an adjuvant strategy to overcome tumor resistance is evaluated.

### 2.3. Targeting NK-1R/SP Axis to Overcome Tumor Resistance

Even though both SP and NK-1R overexpression has a well-established role in carcinogenesis and cancer progression, most strategies targeting NK-1R/SP axis for cancer treatment have focused on receptor inhibition, mainly due to the important role of SP as a neurotransmitter. Furthermore, serine/threonine residues in the NK-1R carboxyl terminal are phosphorylated as a response to the repeated presence of the antagonist, causing a reversible desensitization of the receptor [12,56] after continuous stimulus, SP/NK-1R complexes are endocytosed and internalized, after which NK-1Rs are recycled to the cell surface and SP is degraded [72]. This phenomenon would contribute to a more permanent cell desensitization to SP signaling [56].

One of the main causes why overcoming tumor multidrug resistance remains a major challenge nowadays is its multifactorial nature, which includes both intracellular and multicellular mechanisms, as well as the interaction with the microenvironment, which can potentially protect cells from drug exposure. Therefore, methods designed to overcome multidrug resistance should also be multi-functional, targeting different factors contributing to drug resistance.

Although different agents have been proposed to circumvent tumor multidrug resistance in recent years, most of them have failed in clinical trials due to severe adverse effects. For this reason, and given the proven safety profile of aprepitant, the pharmacological repositioning of this NK-1R antagonist as an adjuvant treatment of resistant tumors is worth exploring, especially when considering the higher rate of NK-1R expression on those tumors with a more advanced stage and worse prognosis [72]. This approach would make perfect sense at a molecular level.

Regarding the contribution of Ca^+2^ signaling in different key processes in cancer, including tumorigenesis, metastasis [61,73] and drug resistance [61,74], preclinical studies have explored the use of Ca^+2^ channel blockers as potential approaches to reverse chemotherapy resistance and immune escape in various malignancies including breast, colorectal, hepatocellular and ovarian cancer [61,75]. However, this approach still faces different drawbacks, such as the lack of specificity of Ca^+2^ blockers or the difficulty in targeting calcium signaling in tumor cells, without affecting normal cells. In this line, the important role of NK-1R activation in regulating Ca^+2^ pathways has promoted studies exploring the effect of NK-1R antagonists, such as aprepitant, on Ca^+2^ signaling in both in vivo and in vitro, showing that they can enhance the efficacy of chemotherapy and help overcome resistance to chemotherapy through the induction of sustained endoplasmic reticulum stress via Ca^+2^ and the consequent suppression of ERK signaling [76]. Indeed, in addition to effectively inhibiting proliferation and preventing the migration of different tumor cells, such as glioma, larynx carcinoma, melanoma and esophageal carcinoma [60,72], NK-1R blockers would induce rapid ER-mitochondrial calcium overload and the accumulation of reactive oxygen species (ROS) in the cell, finally leading to cell apoptosis in both in vitro and in vivo models with NK-1R overexpression [53].

NK-1R inhibition with aprepitant also promotes, in a dose-dependent manner, caspase-dependent apoptotic cell death and G2/M arrest, through inhibiting the PI3K/Akt pathway and its downstream effectors, such as NF-κB, which results in the alteration of the expression of genes involved in drug resistance and cell survival and finally leading to cell apoptosis [77,78,79,80]. Thus, preclinical data suggest that aprepitant would not only be effective as an antiemetic in palliative care, but also as a cytotoxic and anti-proliferative agent against resistant cancer cells, either as a single drug or in combination with chemotherapeutic drugs.

In this respect, since multi-drug resistance is tightly related to metabolic adaptation of tumor cells, resistant cells may also adapt their metabolism to the changes in cytosolic Ca^+2^ concentrations as well as the energetic and oxidative stress caused by NK-1R antagonists. Indeed, similarly, as NF-κB can promote resistance to different chemotherapeutic agents, a recent study has revealed that this transcription factor is also involved in resistance to aprepitant, the reason why the antitumor effect of this NK-1R antagonist could be limited in tumors with an overactivated NF-κB pathway [81] and would require the prior profiling of candidate patients or the need to consider combined use with conventional chemotherapy.

### 2.4. Preclinical Research

After a first in silico screening of approved drugs with potential utility for cancer treatment, and before entering clinical trials in human subjects, drug discovery programs include an experimental stage in which the therapeutic potential of the selected identified molecules are investigated both in vitro and in vivo in specific models.

In recent years, the selective binding of a radiopharmaceutical to a molecular target has been proposed as a valuable tool for the reliable imaging or safe removal of cancer lesions with less side effects [82]. In this respect, recent studies have shown that radiopharmaceuticals based on non-peptide antagonists interact with the corresponding receptor through more binding sites and accumulate better and for a longer time period in cancer cells [82]. Given their low molecular weights, high lipophilicity values and stability, radiopharmaceuticals based on small non-peptide molecules, such as aprepitant, have a great potential for the imaging and therapy of NK-1R-overexpressing cancers [82] and have promoted the development of different NK-1R ligands, labelled with diagnostic or therapeutic radionuclides that are currently being evaluated for their application in targeted radionuclide tumor diagnosis or therapy of NK-1R overexpressing tumors [49,82,83].

To date, the potential utility of NK-1R antagonists as antitumor agents against a wide variety of human malignancies has been demonstrated in different preclinical studies (Table 2), supporting SP/NK-1R system as a valuable predictive factor in cancer and the utility of NK-1R antagonists, such as aprepitant, to promote apoptosis in malignant cells [16].

These studies also show the potential therapeutic value of NK-1R and NK-2R antagonists to treat metastatic disease. Results showing the ability of aprepitant to sensitize tumor cells to arsenic trioxide, vincristine [93], etoposide or doxorubicin [21,94] also provided valuable information about the utility of NK-1R inhibitors to overcome cancer resistance. It should also be emphasized the basic research studies in this field have shown that aprepitant increases the cleavage of poly (ADP-ribose) polymerase (PARP) [77], a nuclear enzyme that repairs DNA damage by adding poly (ADP ribose) polymers and that has been suggested as a promising target for cancer treatment, which may support the use of aprepitant to help to reverse the resistance against PARP inhibitors.

Finally, and contrary to some data suggesting that the inhibitory action of aprepitant would not be specific to tumor cells [87], different studies point to the selective effect of aprepitant on aggressive cancer cell types, mainly due to a lower expression of NK-1 receptors in normal cells [72], but also due to the higher expression levels of the truncated *TACR1* isoform in cancer cells, suggesting the analysis of splice variants as a helpful tool for the stratification of candidate patients for NK-1R targeted therapies [92].

On the basis of these preclinical results with aprepitant, during the past decade different experimental NK-1R antagonists have also proven to present a high affinity for the human NK-1R receptor and to exert a significant antiproliferative activity against human cancer cell lines, such as malignant melanoma, neuroblastoma, glioma, retinoblastoma, pancreas, larynx, gastric and colon carcinoma cell lines [86], which opens the possibilities to new research lines in this field.

### 2.5. Clinical Research

Contrary to the sensitivity to targeted treatments observed in non-resistant tumor cells or in cancer cells with dependency to individual oncogenes that sustain their malignant phenotype (also known as oncogene addiction) [95], multidrug resistant cells are usually characterized by the accumulation of mutations that promote the activation of alternate molecular pathways that circumvent the effect of targeted treatments. For this reason, one of the main strategies to combat drug resistance is the design of combination therapies that target two or more separate pathways at once that do not antagonize each other. In this respect, the use of aprepitant as an adjuvant or combined therapy would be justified, since different studies carried out to date have reported that its co-administration with chemotherapeutic drugs, such as anthracyclines or doxorubicin, can improve the antitumor activity of the drug, while also reducing the severity of the side effects associated with treatment [16,96].

Given its safety profile [16], and as a result of findings in preclinical studies, aprepitant has been proposed as an investigational drug for compassionate use, which is defined as a treatment option for patients with life-threatening, long-lasting or seriously debilitating illnesses that cannot be satisfactorily treated with any other comparable treatment option or are not eligible for any current clinical trial that is using the drug. Specifically, the results of two clinical reports have been published to date: one in a patient with metastatic breast cancer and another one in a patient with metastatic lung cancer:

In the case report published in 2016 by Dr. Lee et al. at St Benedict’s Hospice in Ryhope, UK, the authors reported that the prolonged use of a standard aprepitant dose (80 mg daily during seven months followed by 120 mg every third day) in a patient with metastatic breast cancer suffering from nausea and vomiting, and refractory to all standard antiemetic therapy, with no form of chemotherapy or hormone therapy for 4 months due to her performance status and progressive disease, resulted with a reduction in CA153 tumor marker levels (decreasing from 187 to 122 U/mL) and a good control of nausea/vomiting [97].

More recently, in their case report published in 2019, Dr. Miguel Muñoz´s team in Seville, Spain, showed that aprepitant 1140 mg/day for 45 days was safe and well tolerated by their 76-year-old male patient with a history of chronic obstructive pulmonary disease and diagnosed with non-small-cell (NSCL) squamous cell carcinoma, for whom neither surgical treatment nor chemotherapy was possible. In their work, the team reports that the tumor mass disappeared after patient treatment with aprepitant, suggesting that this NK-1R antagonist could be a good strategy against lung cancer by not only exerting the dual effect of both decreasing the side effects promoted by radiotherapy but also, inhibiting the proliferation of non-small Cell Lung Cancer (NSCLC) cells overexpressing the NK-1R [17].

In addition to breast and lung cancer, since both aprepitant and fosaprepitant are non-peptide NK-1R antagonists with lipophilic properties that can cross the blood–brain barrier and penetrate to the central nervous system [50] without being affected by peptidases, the potential effects of these NK-1R antagonists to prevent or reduce brain metastasis should also be explored.

Finally, and as a result of both preclinical and clinical studies, and in agreement with the European Medicines Agency (EMA) and the USA National Comprehensive Cancer Network to manage cancer patients by including them in clinical trials, on 9 April 2021 a clinical trial (ClinicalTrials.gov Identifier: NCT04840004) started recruiting patients with advanced NSCLC to evaluate the efficacy and safety of a high-dose and long-term use of aprepitant treatment on tumor response, progression-free survival and overall survival [98]. At the time of writing this review, the results of this study have not yet been published.

## 3. Conclusions

Nowadays, resistance to anticancer drugs represents one of the main causes of treatment failure and patient death, thus requiring the development of new treatment strategies to improve the response and prognosis of patients with such tumors.

The increased understanding of the hallmarks of cancer along with the results from different in vitro studies have provided information on the role of the signaling pathways commanded by the axis SP/NK-1R in human cancer and treatment resistance, as well as the potential utility of NK-1R antagonists as antitumor agents that could also be of help to overcome resistance.

During the last years, the repositioning of non-oncology drugs has been suggested as a cost-effective way to overcome the different drawbacks associated with the discovery of new drugs, such as dose-finding and safety profiles, thus facilitating their rapid clinical adoption. Aprepitant is a selective high-affinity antagonist of human SP/NK-1R, which, in addition to having been approved by health authorities for the treatment of chemotherapy-induced vomiting and nausea, has also demonstrated to exert a dose-dependent antitumor effect in both preclinical studies and preliminary results of some case reports.

Given the role of SP/NK-1R in different molecular pathways related to resistance, and even though there is still a need to improve the current knowledge of mechanisms of SP/NK-1R antagonists, such as aprepitant, on resistant cancer cells, studies aimed to evaluate the potential utility of these drugs, either as mono- or as part of drug combination therapies, for the treatment of resistant tumors are justified.

## Figures and Tables

**Table 1:** Main members of the human tachykinin family.

Gene	Tachykinin	Sequence	Preferred Tachykinin Receptor
*TAC1*	Neurokinin 1 (NK1),Substance P (SP)	RPKPQQF**FGLM** [26]	Neurokinin 1 receptor (NK-1R)
	Neurokinin A (NKA) Substance K (SK)	HKTDS**F**V**GLM** [26]	Neurokinin 2 receptor (NK-2R)
	Neuropeptide K (NPK)	DADSSIEKQVALLKALYGHGQISHKRHKTDS**F**V**GLM** [26]	Neurokinin 2 receptor (NK-2R)
	Neuropeptide γ (NP γ)	MKILVALAVFFLVSTQLFAEEIGANDDLNYWSDWYDSDQIKEELPEPFEHLLQRARRPKPQQFFGLMGKRDADSSIEKQVALLKALYGHGQISHKRHKTDSFVGLMGKRALNSVAYERSAMQNYERRR (1st part)GHGQISHKRHKTDS**F**V**GLM** (2nd part) [26]	Neurokinin 2 receptor (NK-2R)
*TAC3*	Neurokinin B (NKB)Neuromedin-K	DMHDF**F**V**GLM** [27]	Neurokinin 3 receptor (NK-3R)
*TAC4*	Endokinin A (EKA)	DGGEEQTLSTEAETWVIVALEEGAGPSIQLQLQEVKTGKASQ**F**F**GLM** [28]	Neurokinin 1 receptor (NK-1R)
	Endokinin A/B (EKA/B)	GKASQ**F**F**GLM** [28]	Neurokinin 1 receptor (NK-1R)
	Endokinin C (EKC)	KKAYQLEHT**F**Q**GL**L [28]	Neurokinin 1 receptor (NK-1R)
	Endokinin D (EKD)	VGAYQLEHT**F**Q**GLL**	Neurokinin 1 receptor (NK-1R)

**Table 2:** Relevant results of preclinical studies.

Cancer Type	Relevant Results
Breast	Compared to non-metastatic cells, metastatic breast cells overexpress NK-1R and NK-2R and are overexpressed in metastatic breast cells compared to non-metastatic cells. Treatment with NK-1R antagonist aprepitant at a 30µM dose enhances promotes Akt phosphorylation, selectively inhibits cell growth and induces cell death in metastatic cells, but not in the non-metastatic 67NR cells [84]. Doxorubicin treatment increases SP levels in both H9C2 cardiomyocytes and MDA-MB-231 triple negative breast cancer cell lines, while pretreating H9C2 cardiomyocyte cell line with aprepitant reduces apoptotic cell death, inhibits oxidative distress and prevents doxorubicin-induced loss of cell viability, compared with doxorubicin alone. On the other hand, MDA-MB-231 pretreatment with aprepitant increases apoptosis, increases the levels of reactive oxygen species and reverses chemoresistance in MDA-MB-231 triple negative breast cancer cells treated with doxorubicin [21]. T47D, BT-474, MDA-MB330, MDA-MB231 and DU4475 human breast cancer cell lines express NK-1R-mRNA and overexpress NK-1 receptors, which are involved in cell viability. While SP induces cell proliferation, treatment with NK-1 receptor antagonists, such as aprepitant, inhibits SP-induced mitogen stimulation and induces cell death by apoptosis through NK-1R [77].
Colon cancer	Aprepitant stimulates the death of SW480 colon cancer cell line by apoptosis and attenuates the Pi3K/Akt signaling cascade. Such treatment also inhibits the NF-ĸB signaling pathway, including the expression of antiapoptotic target genes, without affecting without significant effect on p53 and its downstream proapoptotic p53 target genes [79].
Cervical cancer	SP alters the levels of cell cycle regulators, the expression level of apoptosis-related genes such as BCL-2 and BAX, and enhances migration and proliferation of HeLa cells, which predominantly express the truncated NK-1R isoform. Treatment with NK-1R antagonist aprepitant reverses these effects in a dose- and time-dependent manner [85].
Melanoma	NK-1R is expressed in both human melanoma samples and as well as in MEL HO, COLO858 and COLO679 melanoma cell lines, with a role in tumor cell viability, and treatment with aprepitant in 10–60µM concentrations inhibits cell growth in a concentration- dependent manner by inducing apoptosis [86]. Aprepitant reduces cell viability and proliferation in MeW151, MeW155 and MeW164 melanoma cell lines, although its action is not selective, since aprepitant affects normal cell lines to a similar degree [87].
Lung cancer, Urinary bladder carcinoma	Aprepitant reduces the cell viability and proliferation of E14 human lung cancer and T24 urinary bladder carcinoma cell line, although its action is not selective to cancer cells [87].
Lung cancer	Aprepitant reduces cell viability and proliferation of both normal cells and E14 human lung cancer cell line [87]. NK1-R is upregulated in human and lung cancer samples and is associated with advanced clinical stages and poor prognosis. NK-1R activation promotes cell proliferation, colony formation, epithelial–mesenchymal transition, migration and MMP2/14 expression, while a receptor blockade with aprepitant increases the sensitivity of cancer cells to gefitinib/Osimertinib, inhibits cell proliferation and migration and retards tumor growth in nude mice [63].
Prostate cancer	Prostate cancer cells express the truncated NK1R isoform, and SP affects the expression of cell cycle-related proteins (c-Myc, cyclin D1, cyclin B1, p21) and apoptosis-related genes (BCL-2 and BAX), promoting both proliferative and migrative phenotypes in vitro and stimulating tumor growth in vivo. However, aprepitant administration significantly reverse these effects, enhancing survival time [88].
Glioblastoma	Aprepitant treatment reduces the viability of U87 glioblastoma cell lines in a concentration-dependent manner, and inhibits the oxidizing effects of SP by reducing the production of reactive oxygen species (ROS) and increases the activity of catalase and superoxide dismutase (SOD) [89].
Chronic and acute myeloid leukemia	Aprepitant has strong antiproliferative effect, induces apoptosis and decreases colony formation of K562 and HL-60 cell lines in a concentration-dependent manner [90].
Rhabdoid tumors	NK-1R is overexpressed in both rhabdoid cancer cell lines and human tissue samples of various affected organs, and treatment with aprepitant alone or in combination with cisplatin induces apoptosis and inhibits cell growth [91].
Human pancreatic ductal adenocarcinoma	NK1R inhibition with aprepitant results decreases cell growth in dose-dependent growth reduction in cancer stem cell-like cells (CSCs), parental pancreatic ductal adenocarcinoma (PAC) cells and pancreatic stellate cells (PSCs) in dose-dependent manner. Since aggressive cancer cell types and cell subgroups with higher expression levels of the truncated TACR1 isoform show the highest sensitivity, the analysis of splice variants might potentially be useful of help for the stratification of pancreatic ductal adenocarcinoma (PDAC) patients who are candidates for NK-1R-targeted therapies [92].
